# Understanding ureteropelvic junction obstruction: how far have we come?

**DOI:** 10.3389/fruro.2023.1154740

**Published:** 2023-04-19

**Authors:** Caoimhe S. Costigan, Norman D. Rosenblum

**Affiliations:** ^1^ Division of Nephrology, The Hospital for Sick Children, Toronto, ON, Canada; ^2^ Developmental & Stem Cell Biology Program, Research Institute, The Hospital for Sick Children, Toronto, ON, Canada; ^3^ Department of Paediatrics, Physiology, and Laboratory Medicine and Pathobiology, University of Toronto, Toronto, ON, Canada

**Keywords:** CAKUT (congenital anomalies of the kidney and urinary tract), UPJO, ureteropelvic junction obstruction, development, kidney malformation, pediatric nephrology

## Abstract

Congenital anomalies of the urinary tract are a major cause of chronic kidney disease in both adults and children. Ureteropelvic junction obstruction, usually detected as urinary tract dilatation *in utero*, is one of the most common forms of CAKUT. As antenatal ultrasound technology advances and screening becomes more widespread, increasing numbers of infants with this UPJO will be detected. Management of these infants presents a clinical conundrum, as distinguishing mild benign cases from those who may develop severe renal impairment is challenging. Herein we propose that an understanding of normal developmental and pathological mechanisms involved in UPJO is important in the armamentarium for tackling this challenging condition.

## Introduction

Congenital Anomalies of the Kidney and Urinary Tract (CAKUT) represent the most common congenital anomalies in humans and the largest contributor to childhood chronic kidney disease (1). The prevalence of CAKUT is estimated at 1.6 per 1000 live births, and antenatal hydronephrosis is commonly observed within this group (2, 3). The differential diagnosis for antenatal hydronephrosis is broad. It includes obstructive lesions, classified by anatomical level, from the ureteropelvic junction (UPJ) through the ureterovesical junction (UVJ) to the urethra itself and also non-obstructive lesions of which vesicoureteral reflux (VUR) is the most common (4). There is also a group in which the etiology is somewhat undefined, and urinary tract dilatation represents a benign, self-resolving phenomenon. A non-exhaustive list of causes of antenatal hydronephrosis is included in **Table 1**. Ureteropelvic junction obstruction (UPJO) has been reported to account for up to two-thirds of cases of antenatal hydronephrosis (39-64%) and is recognized as one of the most common forms of CAKUT (5, 6).

**Table 1:** Differential Diagnosis of Antenatal Hydronephrosis.

Causes of Antenatal Hydronephrosis	
Most common	
Transient Hydronephrosis	
UPJ Obstruction	
VUR	
Less common	
UVJ Obstruction	
Posterior Urethral Valves/Urethral Abnormality	
Ureterocele/Ectopic Ureter/Megaureter	
Uncommon	
Prune Belly Syndrome	
Cystic Kidney Disease	

UPJ, ureteropelvic junction; UVJ, ureterovesical junction; VUR, vesico-ureteric reflux.

UPJO can be subcategorized based on the origin of the obstruction, as intramural, mural or extramural. Mural obstruction - a description that refers to an adynamic segment of the ureter, typically with an abnormal distribution of smooth muscle in the ureteric wall – is the most frequently encountered. In this situation, the lumen is not obliterated but narrowed, creating a functional obstruction. Aberrant vessels or adhesive bands causing extramural obstruction can occur but are less common, and in infants, intramural obstruction is rare.

The management of UPJO presents a particularly challenging conundrum for clinicians. While some children will remain asymptomatic and even demonstrate spontaneous resolution of hydronephrosis over time, there are others for whom obstruction heralds significant renal impairment. How to distinguish these groups and determine the best course of management – mainly whether or not to intervene surgically (and if so, when), has been the topic of extensive debate.

With any challenging problem, a return to basic principles is wise. Herein, we will present an overview of what is known about congenital UPJO in terms of molecular mechanisms, pathogenesis, and the pathophysiological consequences of obstruction. We will also provide an overview of the current diagnostic tools at our disposal to decipher clinically which children are at risk of significant renal impairment and what newer options might be on the horizon.

## Normal and abnormal development

The obstructive-recanalization theory of UPJO was one of the first pathogenic theories proposed and was based on observations of human embryos at various stages of development. This theory is based on a descriptive analysis of normal development, indicating that progressive obliteration of the ureter occurred, followed by later recanalization. Failure of this recanalization was proposed to lead to UPJO (7). A subsequent comparative study of rat and human embryos supported to some extent this obstructive-recanalization theory; while the initial obliteration described in the earlier work could, in fact, be observed, this obliteration did not appear to involve the UPJ and hence could not be implicated in the proposed pathogenesis of UPJO (8). This discrepancy highlights the importance of fully elucidating and understanding the normal sequence of developmental events that underlie UPJO to accurately identify pathogenic mechanisms.

## Normal development of the kidney and urinary tract

Human kidney development is dependent on reciprocal inductive interactions between ureteric, stromal, and nephrogenic cell lineages. The ureteric bud (UB) projects from the mesonephric duct into the surrounding metanephric mesenchyme (MM) at approximately the 5^th^ week of human gestation. Ultimately the MM overlying the UB tip will be induced to transition from mesenchymal to epithelial tissue – proceeding through a series of stages of condensation and transition (pre-tubular aggregate -> cap mesenchyme ->renal vesicle-> comma-shaped body->s-shaped body) before finally becoming the functional unit of the kidney, the nephron. Nephrogenesis is complete by 36 weeks of human gestation (normal kidney development is reviewed extensively elsewhere (9)).

The collecting system, that is, collecting ducts, major and minor calyces, renal pelvis, and ureter, develop from the UB lineage. After the initial outgrowth and invasion of the UB, the UB trunk both elongates and undergoes a series of dichotomous subdivisions to ultimately form this collecting system, a process known as branching morphogenesis. The initial branches contribute to the calyces and pelvis, with the extension of the UB trunk caudally toward the cloaca forming the eventual ureter. The intervening pelvic-ureteric junction is part of this continuous epithelial structure and is of ureteric bud origin. However, the origin of the surrounding mesenchyme diverges at the site of the UPJ. The mesenchyme surrounding the tubular portion of the UB is tail-bud mesenchyme and is distinguished from the metanephric mesenchyme by the expression of the transcription factor *Tbx18 and Tsh3* (10). It appears to be this distinction in surrounding mesenchyme that determines this caudal portion of the UB patterning into the ureter rather than collecting duct apparatus. Hence the UPJ is a site of differential mesenchymal cell contribution, which may be relevant to the pathogenesis of obstruction here (10, 11).

The molecular events governing ureteric bud induction and the pathways involved in initial branching morphogenesis are described in a comprehensive review by Blake and Rosenblum (12). The normal sequence of events in the development of the ureter proper has also been interrogated (8, 13). If and how these events differ at the site of the UPJ, making it specifically vulnerable to pathological obstruction, is less clear.

In the developing ureteric shaft, two cell types predominate; an inner epithelial cell layer and overlying mesenchyme – reciprocal interactions between these layers are critical for the normal formation of the mature functioning ureteric structure. The inner epithelial layer proliferates and differentiates into what will ultimately become the transitional epithelium or urothelium. Both paracrine signaling from this epithelium and autocrine signaling from the mesenchyme, itself, drive smooth muscle cell (SMC) differentiation along with differentiation of stromal and adventitial fibroblasts (14). The SMC layers will ultimately be arranged into an inner longitudinal layer and an outer circular layer. The transition from mesenchyme to SMC begins around the 12^th^ week of human gestation, suggesting that although the hydronephrotic consequences of UPJO are often not appreciated until later gestation (second trimester, most often), the embryological disruption may occur much earlier (15).

The ultimate function of these smooth muscle layers is to allow the coordinated sequential contraction of the ureter and facilitate normal urine flow (15). Failure of this peristaltic mechanism leads to the urinary stasis, accumulation, and ultimately, retrograde pressure, seen in UPJO. Two pacemaker cell (PMC) populations orchestrate this process and hence have been a focus of interest in UPJO pathogenesis. The first population of PMCs are located at the pelvic-kidney junction and are marked by the expression of hyperpolarization-activated cyclic nucleotide-gated channel 3 (HCN3) and low voltage-gated T-type calcium channel (CaV 3.1) (15–17) . The other pacemaker population consists of Cajal-like cells (CLCs), which are spread throughout the ureter but are believed to be at highest concentration at the UPJ (18). Spontaneous peristalsis in a denervated ureter has been demonstrated during observation of explanted ureters *in vitro* and is also seen in the function of the ureter in kidney transplantation *in vivo*. These observations suggest that this sequential contraction is a myogenic rather than a neurogenic process, again suggesting a smooth muscle defect is likely to be implicated in situations where this peristaltic machinery fails.

**Figure 1:**
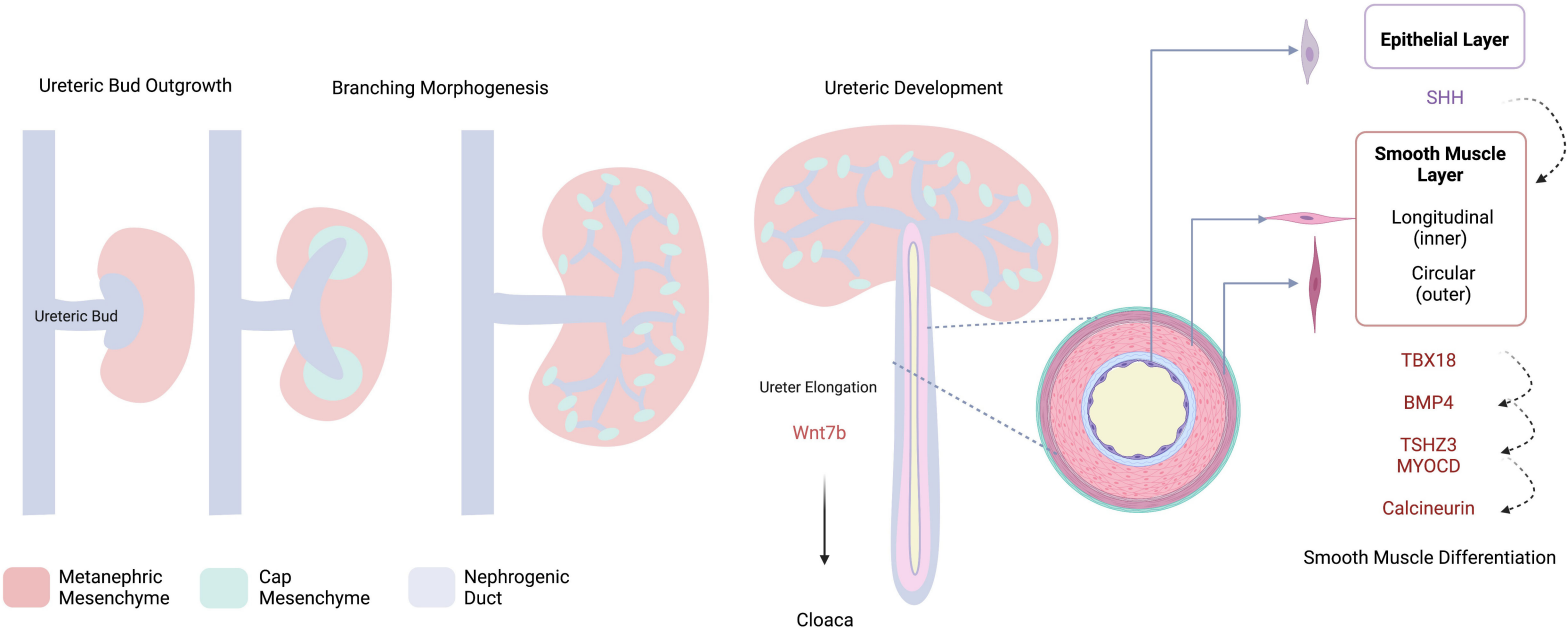
Schematic Overview of Collecting System Development. The ureteric bud invades the metanephric mesenchyme, cap mesenchyme is induced via a number of reciprocal interactions and will ultimately form the nephron. Dichotomous branching of the UB contributes to the development of the collecting system from which the developing ureter elongates towards the cloaca. Development of the ureter – smooth muscle and epithelial layers- is controlled by reciprocal interactions involving a number of molecular drivers; SHH, sonic hedgehog; TBX18, t-box transcription factor 18; BMP4, bone morphogenic protein 4; TSHZ3, teashirt zinc finger homeobox 3, MYOCD, myocardin. Created with BioRender.com.

Acknowledging this mesenchymal junction as a potential nidus of vulnerability, in addition to recognizing the importance of smooth muscle development in normal ureteric function, it follows that dysregulation of any of the pathways involved in mesenchymal differentiation may be implicated in UPJO pathogenesis. A number of animal models of UPJO have been developed on the basis of this hypothesis, which we will discuss in a later section of this review. **Figure 1** outlines some of the processes involved in normal collecting system and ureteric development.

## Pathogenesis of UPJO

In 1942, Karl Ostling, a Swedish urologist, in his thesis “The Genesis of Hydronephrosis – particularly with regard to the changes at the ureteropelvic junction,” stated that “In spite of the profuseness of the literature on the subject … a great many problems in this field remain unsolved” in particular, “want of an elucidation of the genesis [of hydronephrosis] … as a prerequisite for therapy” (19). Some 70 years later, the pathogenesis of UPJO remains largely undefined, and the desire to understand this complex entity persists.

**Figure 2:**
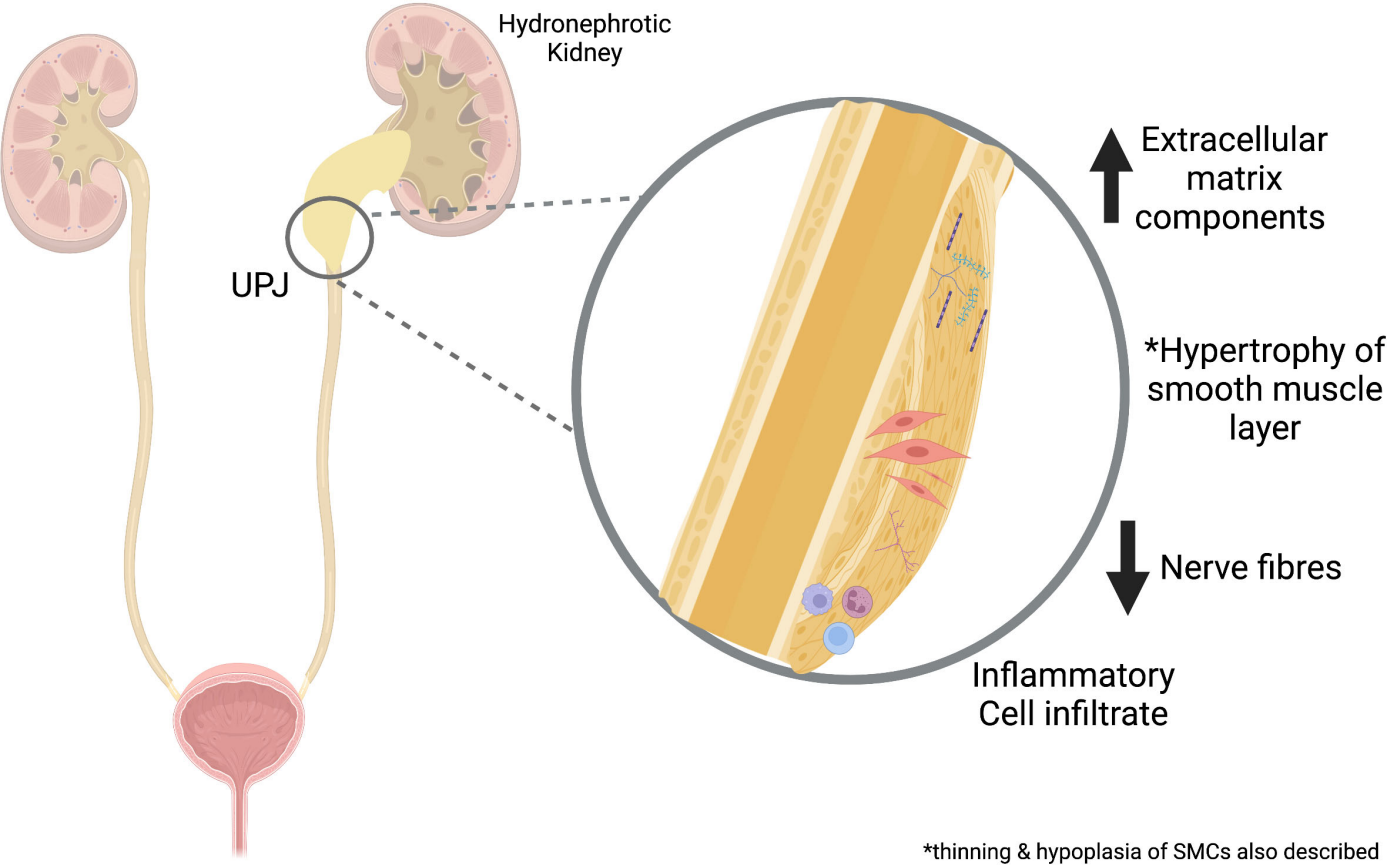
Overview of Pathological Changes in UPJO. Proposed mechanisms involved in UPJO pathogenesis include increased extracellular matrix, smooth muscle changes, inflammatory cell infiltrates, and reduced nerve fibers. UPJ, ureteropelvic junction obstruction. Created with BioRender.com.

Several studies have characterized the histopathological changes at the UPJ using both animal models and human samples excised at the time of pyeloplasty. Gross observations have included smooth muscle hypertrophy, perifasicular fibrosis, and inflammatory cell infiltration, with a notably intact epithelial layer (20, 21). Muscular hypoplasia has also been described with thin muscle fibers, dense interwoven collagenous network, and reduced neural components (22). Extracellular markers – including fibronectin, type IV collagen, and laminin, were all found to be increased within the smooth muscle compartment and stromal matrix in human UPJO samples (23). This suggested a role for the over-deposition of collagen and an increased collagen-to-smooth muscle ratio which has been a consistent finding and has been attributed to the malfunction of smooth muscle cells (20, 22, 24). Generally, these histopathological findings support mesenchymal dysregulation in the pathogenesis of UPJO. **Figure 2** provides a schematic overview of some of these pathological changes.

A number of murine models have shown findings consistent with this myogenic theory and, in particular, have suggested a role for dysregulated TGF-β signaling in the pathogenesis of UPJO. Bmp4, a member of the TGF-β superfamily, is highly expressed in both metanephric and tailbud-derived mesenchyme. Bmp4 signaling appears to have an autocrine effect, promoting differentiation of smooth muscle, in part through upregulation of *TSHZ3*. It is also recognized to have a role in ureteric growth and elongation (11). Bmp4 signaling is critical for ureter formation in the mouse and, when decreased, results in a dose-dependent loss of ureteral smooth muscle formation and the development of UPJO (11, 25). *BMP4* variants have been detected in humans with UPJO but are rare (26).

Smad4 is critical to the transcriptional response to TGFβ signaling, including Bmp4, and hence is also important in normal ureteric development. Mice with conditional knockout of *Smad4* in the ureteral mesenchyme have differentiated SMCs, but both the quantity and contractility of these cells are reduced. Ureteric peristalsis is maintained but uncoordinated. Functional obstruction occurred early, with physical distortion of the ureter and persistent structural obstruction later. Interestingly the UPJO phenotype seen in these Smad4 mutants was not as severe as that in isolated BMP4 mutants, suggesting that some actions of Bmp4 may be smad-independent in ureteral smooth muscle (27, 28). Transcriptomic analysis showed reduced expression of Bmp4 downstream effectors, including *Id2*. Independently, *Id2 deficient* mice demonstrate hydronephrotic phenotypes, hence supporting the central role of this pathway in UPJO pathogenesis (29).

Sonic hedgehog (SHH) is expressed by the ureteric epithelium and surrounding stromal cells and induces mesenchymal proliferation and SMC differentiation through its interaction with the *Patched1* receptor *(ptchd1)*. SHH induces the expression of Bmp4, a member of the TGFβ superfamily (30) Dysregulated hedgehog (HH) signaling has been implicated in the pathogenesis of UPJO. Constitutive activation of hedgehog-GLI3 signaling in mice induced an ectopic population of cells characterized by cortical stromal markers that obstruct the UPJ. This was correlated with similar findings of molecular characteristics of stomal progenitors and active hedgehog signaling in the UPJ specimens from children at the time of pyeloplasty, suggesting that activated hedgehog-GLI signaling is part of the pathogenesis of UPJO. Interestingly in this model, disruption of the ureteric smooth muscle was not observed; hence a distinct pathogenic mechanism implicating stromal cell differentiation is proposed (31).

The peristaltic mechanism which guides urine flow may also be implicated in UPJO pathogenesis. Miyazaki et al. generated mutant mice lacking the angiotensin 1 (AT1) receptor. They noted that the renal pelvis was completely absent in these mice, and smooth muscle differentiation throughout the ureter was significantly reduced. Peristaltic activity is proposed to be initiated at the renal pelvis; in the mutant mice without a renal pelvis, peristaltic waves were absent (32). Chang et al. generated mice with selective deletion of *cnb1* in the developing ureteric mesenchyme, disabling lineage-specific calcineurin function. They also demonstrated compromised SMC proliferation, abnormal renal pelvis formation, and altered peristalsis leading to progressive obstruction, with a phenotype mimicking UPJO in humans (33).

Several other genes have been implicated in UPJO pathogenesis, predominantly based on findings of abnormal mesenchymal differentiation in mouse models of UPJO. These include *Adamts-1, Agt, Agtr1a/b, Aq2, Calcineurin, Id2, Nfa, TBX18* and *Tshz2 and 3* (10). Interestingly, despite steadily advancing genetic sequencing techniques and increased availability of genetic testing, monogenic causes of UPJO, specifically, have yet to be consistently found in humans. *TSHZ 2/3* variants, although strongly implicated in mouse models, were not found to be a cause of UPJO in a small human cohort (14, 34). Heterozygous variants of *TBX18* have been associated with human CAKUT, including UPJO (35). In most cases, a monogenic cause is not identifiable, suggesting that no single molecular pathway is responsible but rather a concerted action of several factors, and hence most likely a polygenic etiology. A recent systematic review that examined genetic factors in UPJO identified 15 differentially expressed genes in UPJO. There was an association with pathways related to hypoxia, fibrosis, and inflammation in developing this condition – pointing to a need for further studies of these pathways in understanding the genetic mechanisms that control the genesis of UPJO (36).

## Consequences of urinary tract obstruction

The debate regarding the management of UPJO depends on the understanding that untreated urinary tract obstruction endangers renal function. Although this statement is broadly true, it is nuanced; timing, duration, and extent of the obstructive lesion are all important variables to consider. Additionally, with congenital UPJO, the insult is likely two-fold, reflecting both obstructive and developmental injury processes (37).

Surgical ligation of the ureter or genetic manipulation in animal models has allowed the study of the impact of various models of obstruction at a pathological level. In humans, nephrogenesis is complete by approximately 36 weeks gestation. In mice and rats, renal development persists until the third postnatal day. Thus, neonatal mice are a viable model for congenital UPJO (38, 39). Other animal models of unilateral ureteral obstruction (UUO), such as sheep, require obstruction of the ureter *in utero* to accurately reflect human development (5).

A number of early studies of unilateral ureteral obstruction were conducted in second-trimester lamb fetuses and described histological consequences consistent with renal dysplasia (40–42). Findings included reduced glomerular number, abundant but disorganized stroma, and cyst formation. A detrimental effect on kidney growth and nephrogenesis was also observed in both murine and porcine models (37, 38, 43). Similar dysplastic findings have been identified in kidney biopsy specimens in humans with UPJO (21, 42).

The benefits of early decompression were supported by some of these early animal models of obstruction. Harrison et al. noted that early functional recovery was inversely proportional to the duration of the obstruction in the fetal lamb model, while Chevalier and colleagues came to a similar conclusion based on work in mice (44, 45). Others, however, have found that histological and functional renal damage may persist despite relief of obstruction, even when conducted during early stages of urinary tract development (46, 47).

When renal development is more mature, the mechanism of injury may be attributed to obstructed urine flow leading to increased intraparenchymal pressure. This retrograde pressure precipitates tubular stress and altered perfusion, triggering a cascade of inflammatory and oxidative events. Tubulointerstitial injury is seen in both human biopsy specimens and animal models of obstructive uropathy. Increasing hydrostatic pressure drives progressive tubular cell swelling, with sheer and oxidative stress, leading to altered cellular metabolism and apoptosis (48). There is activation of the intra-renal renin-angiotensin-aldosterone system activating vasoconstrictive mechanisms and propagating injury (5). Tubular damage has been reported to disproportionately affect the distal and collecting tubules, although proximal tubular changes are also seen. Down-regulation of both vasopressin receptors and aquaporins in the collecting duct is seen in both human and animal models (49). Functionally, tubular damage impairs the ability to modify sodium and water reabsorption and urinary acidification, correlating clinically with the polyuric, salt-losing phenotype seen in many children with congenital obstructive uropathy (15).

Interstitial changes are characterized by inflammatory cell infiltration with an array of cytokines implicated in propagating the inflammatory and oxidative injury cycle (50). Increased angiotensin II expression is implicated, in part, in initiating oxidative damage and promoting fibrosis, whilst antioxidant mechanisms such as nitric oxide production are downregulated (51). Tubulointerstitial fibrosis is the final common pathway in renal injury and is potentiated by both resident and migratory myofibroblasts producing TGFβ and TNF-α among other profibrotic cytokines. A number of reviews have interrogated these processes in more detail (37, 43). Fibrotic change is typically irreversible, and as in other forms of kidney injury, the degree of fibrosis typically (although not always) correlates with the extent of renal impairment. (Han et al., 1998, Zhang, 2000).

Unfortunately, the time course of this injury pattern and hence the potential window of recoverability remains unclear. In addition, how extensive an obstructive lesion must be (and how well this is captured by the current assessment tools) to trigger these events is uncertain. This ambiguity again highlights the difficulty of clinical care decisions.

Broadly, it appears that persistent ureteric obstruction, if established early in development, may impede normal nephrogenesis leading to dysplastic changes and reduced nephron number. This already reduced reserve of functional kidney tissue is then potentially exposed to further risk of injury with unrelieved obstruction. In this context, it is imperative to recall that the nephron number is determined by 36 weeks of gestation. Thereafter, although adaptive changes are possible, no mechanism exists to replace damaged nephrons (52).

Factors that alter nephron endowment, such as premature birth, or intrauterine growth restriction, further increase the risk associated with ureteric obstruction. This has been demonstrated in murine models, where mice with a lower nephron mass were more susceptible to further nephron loss, and nephron growth was not restored with relief of the obstruction as occurred in wild-type mice (53).

In addition, when nephron mass is reduced – for any reason – increased demand is placed on the residual nephron contingent. Over time, hyperfiltration can herald glomerular injury in the remaining functional nephrons with progressive sclerosis and eventual nephron loss, driving long-term renal dysfunction (54).

It has been established that even children with milder forms of CAKUT, and apparently normal renal function, can develop chronic kidney disease in adulthood, with a proportion requiring kidney replacement therapy (1, 55). Hence, although controversy remains about optimal surgical management, longer-term surveillance and nephroprotective strategies may also be pertinent and should be considered in some patients.

## Assessment of UPJO

The management of UPJO has become progressively more conservative over recent years. Most patients avoid surgical intervention and can be safely evaluated with serial ultrasonography and functional imaging as needed (usually done if the ultrasound suggests deterioration). It has been shown that even children with severe hydronephrosis initially can demonstrate resolution over time – 25% showing improvement or stabilization with mild dilatation, 50% with moderate, and the remaining 25% with persisting severe dilatation over the first two years (56, 57). A large meta-analysis of over 1000 children supported that 80% of those with unilateral UPJO can be managed conservatively and safely (58). However, a significant proportion of children who are initially managed conservatively will go on to need surgical intervention; 25%, according to the aforementioned meta-analysis, and over 50% in a large retrospective cohort (59).

The decision to intervene is generally based on an assessment of the impact of the obstruction on renal function and extrapolation of the risk of progressive loss of function if left untreated. Specific indications vary between institutions but typically rely on a combination of ultrasound, functional imaging, and serum markers of renal function, in addition to clinical features such as urinary tract infections or urolithiasis (4). Unfortunately, the diagnostic tools, which are heavily relied upon to inform the decision-making process, have several limitations, particularly in an infant population.

Generally, “renal function” is perceived to be synonymous with glomerular filtration rate (GFR), and serum creatinine is the laboratory marker used to estimate this. This has limitations, particularly in infants in whom assessment of GFR is particularly challenging. Transplacental passage of maternal creatinine, size, muscle mass, and hydration level may all impact measurement. Additionally, creatinine can be initially normal, even if there is a significant nephron loss, due to compensatory filtration of the remaining nephron mass at that time. Hence a normal serum creatinine should not be entirely reassuring. Unfortunately, no superior measurement is currently recommended, with cystain c and other serum biomarkers, also subject to limitations.

Regarding radiological assessment, ultrasonography is the first line. It is inexpensive, non-invasive, and widely available. UPJO, initially identified as urinary tract dilatation, is usually detected on second-trimester ultrasonography. These antenatal images can provide information on laterality, the extent of dilatation, and associated abnormalities such as abnormal renal size, parenchymal appearance (cystic or echogenic), or oligohydramnios, which may portend a less favorable prognosis. Despite improving sonographic technology and skill, some children still evade antenatal detection and may present symptomatically or be detected on post-natal screening for other indications. An initial post-natal ultrasound can be falsely reassuring in cases of UPJO if the hydration status of the infant is suboptimal. Inadequate urine volume can mask urinary tract dilatation. Hence it is recommended to wait 48 – 72 hours before the initial ultrasound.

Various grading systems exist for quantifying the severity of obstruction - most prioritize anteroposterior diameter (APD) of the renal pelvis. Others have demonstrated that combining APD and the degree of caliectasis may be more useful in distinguishing children with moderate hydronephrosis at risk of surgical intervention (60–62). No single system is universally agreed upon.

Radionucleotide or functional imaging has an important role in urinary tract obstruction. ^99m^Tc-mercaptoacetyltriglycine (MAG-3) is the most commonly used radionucleotide in children with suspected UPJO. MAG-3 is injected intravenously and extracted from the plasma by proximal tubular cells, where it accumulates and is then secreted into the tubules for excretion. It is proposed that the tracer uptake or plasma extraction rate provides an estimate of relative renal function (<40% considered abnormal), and the washout phase reflects the degree of obstruction (T1/2 < 10mins considered normal, >20 mins considered abnormal). MAG-3 is affected by GFR and hence is not as reliable in younger infants with a naturally lower GFR. It is typically deferred until 2-3 months of life for this reason (63). Unfortunately, the correlation of pelvic dilatation with radionucleotide imaging is best in severe cases, with most moderate cases being less reliable (64). Serial comparison may, however, be useful and is often used in the decision-making process when surgical intervention is considered. However, this modality relies on the assessment of relative renal function. Thus, compensatory hypertrophy of the contralateral side could alter the result without an actual decline in renal function on the affected side.

Recently, machine learning has been applied to MAG-3 renal scans in an effort to predict clinical outcomes in children with antenatal hydronephrosis. A deep learning model was able to predict children at increased risk of renal complications based on analysis of radiotracer concentration against time in a case-control study (65). Although this work is early, it highlights a potential role for artificial intelligence to augment the currently available tools in assessment of UPJO, in the not so distant future.

Better ways to identify children who would benefit from surgical intervention are clearly needed. To this end, a large number of urinary biomarkers have been explored; (TGF-β, TNF-α, N-acetyl-β-D-glycosaminidase, Epidermal Growth factor (EGF), CCL2, angiotensinogen, Prostaglandin reductase 1 (PTGR1), Ficolin-2, Nicotinate-nucleotide pyrophosphorylase, Immunoglobulin superfamily containing leucine-rich repeat protein (ISLR), vascular cell adhesion molecule-1 (VCAM-1), metalloproteinases (MMP), anti-microbial peptides, and polyomavirus (66–75)). Recently a combination of an MMP-7 and tissue inhibitor of MMP -2 (TIMP-2) was found to be predictive of reduced renal function in children with UPJO. The combination has been proposed to have the potential to assist with surgical decision-making in the future (76).

Metabolomics is an exciting field in biological investigation, which has recently been applied to UPJO. Metabolic profiling using nuclear magnetic resonance (NMR) spectroscopy was performed on infants with antenatal hydronephrosis and healthy controls. The technique distinguished patients with UPJO requiring surgery from those with transient dilatation and healthy controls, making this an exciting prospect for the future (77). As with the emerging use of biomarkers in other fields, in addition to the need to demonstrate precision and reproducibility, limitations in the transition from bench to bedside lie in accessibility, cost, and ease of interpretation. However, it is an advancing field and certainly sparks optimism for future non-invasive diagnostic options for UPJO.

## Conclusion

The long-term outcome is generally considered favorable for children with UPJO; however, this is based on relatively few long-term studies. Increasing recognition that children with milder forms of CAKUT, and apparent normal renal function, are being identified in adulthood with chronic kidney disease is concerning. However, determining which children are likely to follow this trajectory, particularly within the spectrum of UPJO, is challenging. In some children with severe disease, in particular, those who undergo pyeloplasty, it is clear that long-term follow-up is warranted, while in those with moderate or conservatively managed UPJO, the optimum strategy is not as certain (78). Regarding the etiology of UPJO, it seems that despite advances in disease modelling, molecular biology, and genetics, the pathogenesis of this condition remains largely undefined. Defining pathogenic mechanisms driving this condition is likely critical to developing disease-specific diagnostic and therapeutic strategies.

## Author contributions

CC drafted the article, including the creation of figures. NR provided critical revision of the article and provided final approval of the version to be published.
